# Spatial organization of bacterial sphingolipid synthesis enzymes

**DOI:** 10.1016/j.jbc.2024.107276

**Published:** 2024-04-07

**Authors:** Chioma G. Uchendu, Ziqiang Guan, Eric A. Klein

**Affiliations:** 1 Center for Computational and Integrative Biology, Rutgers University-Camden, Camden, New Jersey, USA; 2 Department of Biochemistry, Duke University Medical Center, Durham, North Carolina, USA; 3 Biology Department, Rutgers University-Camden, Camden, New Jersey, USA; 4 Rutgers Center for Lipid Research, Rutgers University, New Brunswick, New Jersey, USA

**Keywords:** sphingolipid, ceramide, lipid metabolism, microbiology, subcellular localization

## Abstract

Sphingolipids are produced by nearly all eukaryotes where they play significant roles in cellular processes such as cell growth, division, programmed cell death, angiogenesis, and inflammation. While it was previously believed that sphingolipids were quite rare among bacteria, bioinformatic analysis of the recently identified bacterial sphingolipid synthesis genes suggests that these lipids are likely to be produced by a wide range of microbial species. The sphingolipid synthesis pathway consists of three critical enzymes. Serine palmitoyltransferase catalyzes the condensation of serine with palmitoyl-CoA (or palmitoyl-acyl carrier protein), ceramide synthase adds the second acyl chain, and a reductase reduces the ketone present on the long-chain base. While there is general agreement regarding the identity of these bacterial enzymes, the precise mechanism and order of chemical reactions for microbial sphingolipid synthesis is more ambiguous. Two mechanisms have been proposed. First, the synthesis pathway may follow the well characterized eukaryotic pathway in which the long-chain base is reduced prior to the addition of the second acyl chain. Alternatively, our previous work suggests that addition of the second acyl chain precedes the reduction of the long-chain base. To distinguish between these two models, we investigated the subcellular localization of these three key enzymes. We found that serine palmitoyltransferase and ceramide synthase are localized to the cytoplasm, whereas the ceramide reductase is in the periplasmic space. This is consistent with our previously proposed model wherein the second acyl chain is added in the cytoplasm prior to export to the periplasm where the lipid molecule is reduced.

Sphingolipids play a critical role in eukaryotic cells where they are involved in membrane structure and function and serve as important signaling molecules (1). By contrast, prokaryotic sphingolipids were considered to be rare, with notable exceptions in Bacteroidetes where they play a role in modulating the host immune response (2) as well as Sphingomonads where they functionally replace lipopolysaccharide (LPS) (3). One reason that bacteria were thought to lack sphingolipids is that, apart from serine palmitoyltransferase (Spt), they do not encode homologues of the synthetic enzymes used in eukaryotes.

In a study of adaptation to phosphate limitation in *Caulobacter crescentus*, we found that this organism is also capable of producing sphingolipids (4). Using a set of genetic screens, we and others identified the key genes required for bacterial sphingolipid synthesis (5, 6) (Fig. 1). In addition to the conserved *spt* gene, we identified genes encoding a reductase (*cerR*) and an acyltransferase (*bcerS*). Our biochemical data, along with bioinformatic analyses, led us to propose a synthetic pathway in which addition of the second acyl chain occurs prior to lipid reduction (Fig. 1) (6), which is distinct from the well-characterized eukaryotic pathway. However, others in the field have proposed that these enzymes do, in fact, perform this synthesis in a pathway akin to eukaryotes (Fig. 1) (5).

**Figure 1:**
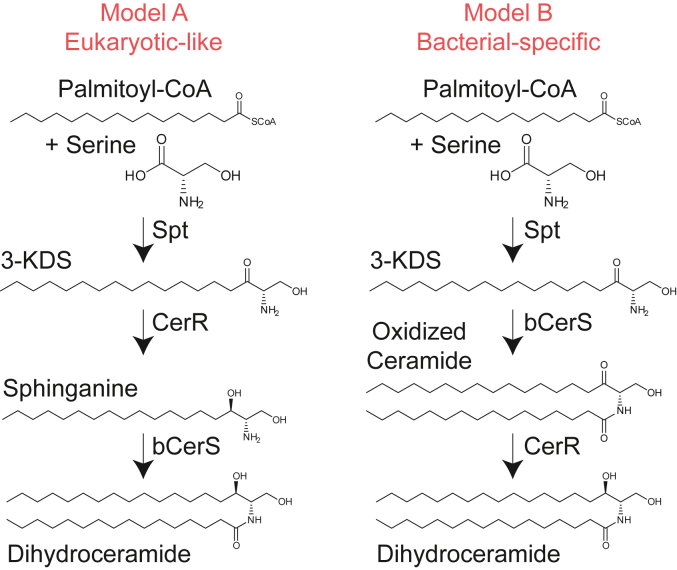
**Proposed mechanisms of bacterial sphingolipid synthesis.** Two recent studies identified the genes required for ceramide synthesis in *C. crescentus* (5, 6). The proposed synthetic mechanisms either follow a similar chemistry to that found in eukaryotes (*left*) or operate in a bacterial-specific order (*right*).

To distinguish between these two proposed models, we have investigated the spatial organization of the sphingolipid synthesis enzymes. Using a set of orthogonal experimental approaches, we found that Spt and bCerS are cytoplasmic proteins, whereas CerR resides in the periplasm. Given that these lipids are ultimately trafficked to the outer membrane (7, 8), our findings support the model in which Spt and bCerS act first in the cytoplasm prior to lipid translocation to the periplasm for subsequent reduction and trafficking to the outer membrane.

## Results

### Cell permeabilization reveals the spatial arrangement of sphingolipid synthesis enzymes

**Figure 2:**
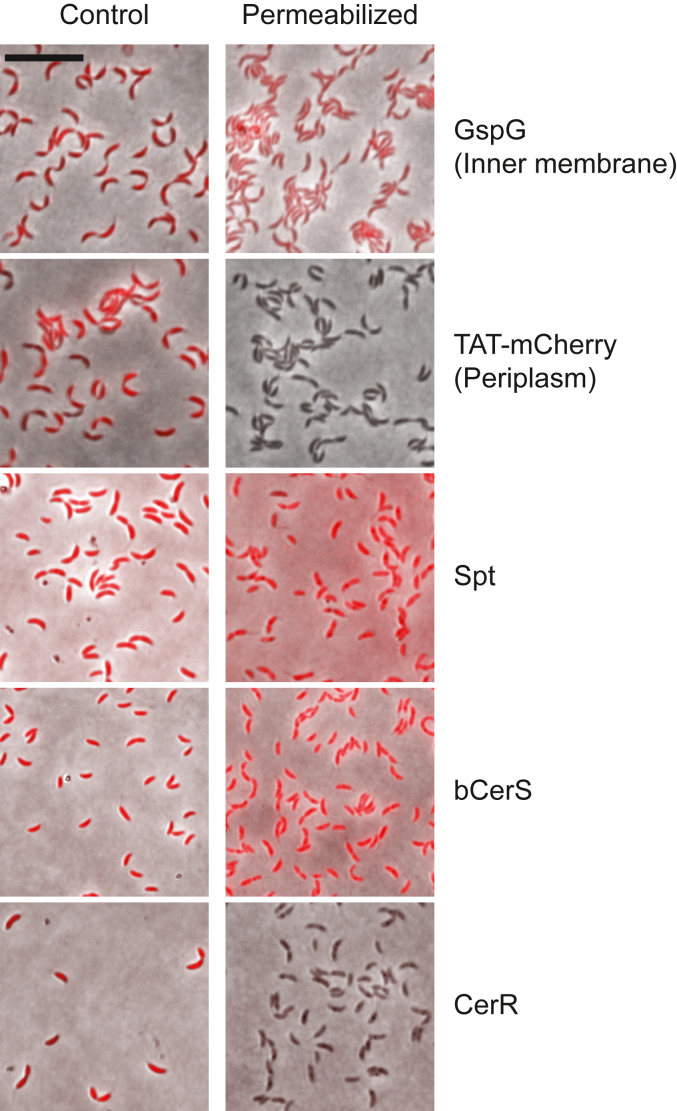
**Permeabilization of the outer membrane suggests distinct subcellular localizations of the sphingolipid synthesis enzymes.** Cells expressing the indicated mCherry-tagged proteins were grown overnight in the presence of 0.3% xylose or 0.5 mM vanillate to induce expression. GspG-mCherry (10) and TAT-mCherry (9) are control inner-membrane and periplasmic proteins, respectively. Control and permeabilized cells were visualized by fluorescence microscopy and the loss of fluorescence upon permeabilization was assessed. The images are the overlay of phase and fluorescent images. Scale bar = 10 μm.

Our previous mass spectrometry analysis of sphingolipid intermediates demonstrated the accumulation of oxidized ceramide upon *cerR* deletion (6). The presence of a second acyl chain in this molecule suggests that either CerR acts after the addition of the second acyl chain, or bCerS acylates both 3-ketodihydrosphingosine (oxidized long-chain base) and sphinganine (reduced long-chain base). To distinguish between these two possibilities, we considered that these enzymes may occupy distinct subcellular niches. To assess protein localization, we repeated a previously reported experiment and expressed mCherry-tagged alleles of Spt, bCerS, and CerR and incubated the respective bacteria with chloroform-saturated Tris buffer, which preferentially permeabilizes the outer membrane and releases soluble periplasmic proteins (6, 9). Spt and bCerS retained fluorescence upon permeabilization, indicating their potential localization in either the cytoplasm or inner membrane (Fig. 2). By contrast, CerR showed a complete loss of signal, suggesting that it is most likely a soluble periplasmic protein (Fig. 2). These results are consistent with our hypothesis that these enzymes are compartmentalized differently within the cell.

### Beta-lactamase fusion assay to evaluate periplasmic protein localization

**Figure 3:**
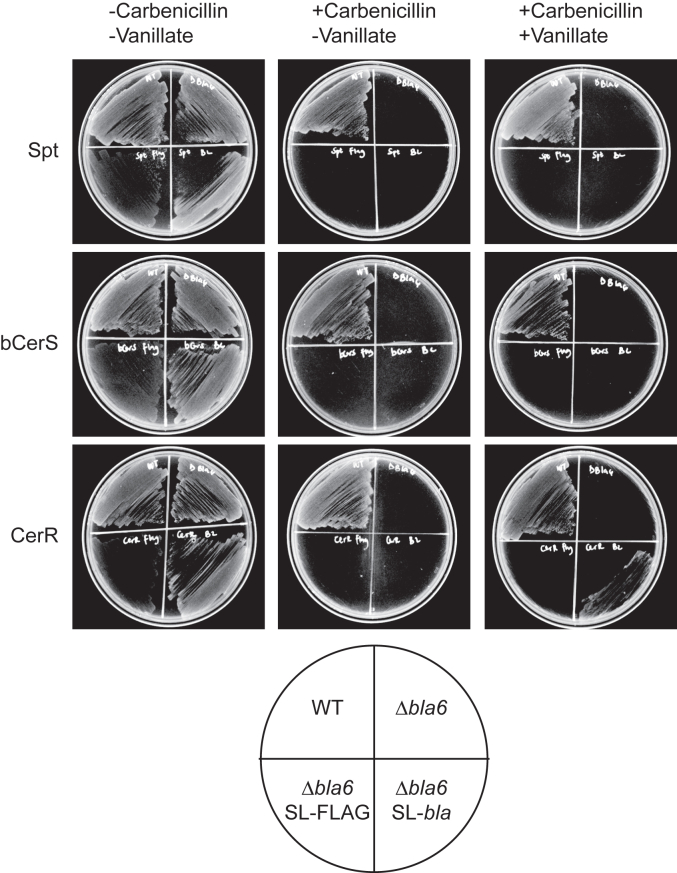
**Beta-lactam resistance indicates periplasmic localization.** The indicated strains were grown overnight ±1 mM vanillate to induce beta-lactamase fusion expression. Wild-type (carbenicillin resistant) and Δ*bla6* (carbenicillin sensitive) cells were included as controls. The strains were streaked onto PYE agar plates as diagramed and growth was assessed after 48 h. Plates were either plain PYE (all strains should grow), PYE + carbenicillin (only WT should grow), and PYE + carbenicillin + vanillate (only WT and periplasmic beta-lactamase fusions should grow).

As an orthogonal approach to assessing subcellular localization, we utilized beta-lactamase (bla) fusions as a probe for periplasmic secretion (10). The mechanism of action of beta-lactam antibiotics, such as carbenicillin, involves the inhibition of penicillin-binding proteins (PBPs) in the periplasm of Gram-negative bacteria (11). To test for periplasmic localization, we made vanillate-inducible bla-fusions to the C-termini of Spt, CerR, and bCerS. We chose C-terminal fusions to avoid potentially disrupting any N-terminal signal sequence required for protein secretion. Additionally, *C. crescentus* is naturally resistant to carbenicillin due to the expression of beta-lactamase (12); therefore, we conducted all experiments using the *bla6* deletion strain (13) to restore beta-lactam sensitivity. Finally, the fusion constructs lacked the *bla* signal sequence to ensure that any secretion was due solely to the sphingolipid-synthesis enzyme of interest. The respective strains were cultured both in the presence and absence of vanillate and/or carbenicillin. Growth of wild-type and Δ*bla6* strains on carbenicillin plates confirmed their respective antibiotic sensitivities (Fig. 3). In the presence of both carbenicillin and vanillate, only the CerR fusion displayed growth (Fig. 3), whereas Spt and bCerS fusions remained sensitive to carbenicillin (Fig. 3). By contrast, expression of the FLAG-tagged enzymes did not restore carbenicillin resistance. These results are consistent with the data obtained by permeabilizing the outer-membrane (Fig. 2) and support the periplasmic localization of CerR.

### Solubility of the sphingolipid synthesis proteins

**Figure 4:**
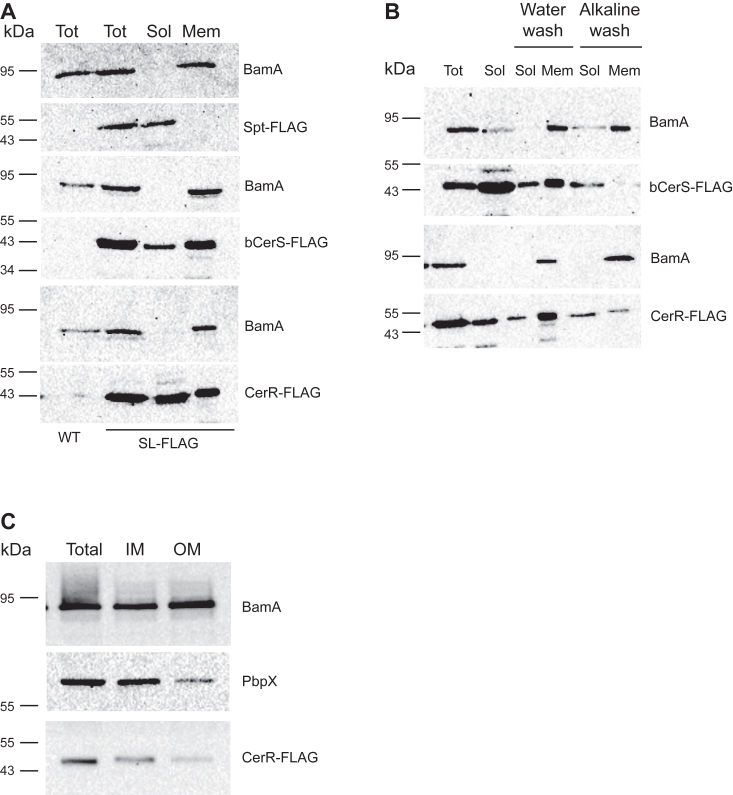
**Subcellular fractionation identifies soluble and membrane-bound proteins.** Cells expressing FLAG-tagged sphingolipid synthesis enzymes were grown overnight with 0.5 mM vanillate to induce expression. *A*, cells were lysed *via* French press and membranes were collected *via* ultracentrifugation. Total cell lysates (Tot), soluble proteins (Sol), and membrane proteins (Mem) were resolved by SDS-PAGE and analyzed by Western blot. BamA served as a membrane control protein. Wild-type (WT) cells did not express a FLAG-tagged enzyme that serves as an anti-FLAG negative control. *B*, membrane pellets collected as above were washed with either water or an alkaline solution (0.1 M Na_2_CO_3_, pH 11) to release peripheral membrane proteins. Samples were analyzed by Western blot as above. *White gaps* indicate where the membranes were cut for antibody incubation. *C*, membranes from cells expressing CerR-FLAG were separated on a sucrose gradient. Total, inner, and outer membrane fractions were analyzed by SDS-PAGE. Antibodies against BamA and PbpX were used as controls for the outer and inner membranes, respectively.

The eukaryotic Spt, KDSR, and CerS enzymes are all intrinsic membrane proteins (14). The previous characterization of the bacterial Spt demonstrated that, by contrast, this enzyme is soluble (15). To determine the solubility of CerR and bCerS, we performed subcellular fractionation assays to separate soluble and membrane-associated proteins. Our analysis confirmed that Spt is soluble (Fig. 4*A*). By contrast, both bCerS and CerR were found in both the soluble and membrane fractions (Fig. 4*B*). Analysis of these protein sequences with CCTOP did not identify any predicted transmembrane regions (16); therefore, we questioned whether these proteins are integral or peripheral membrane proteins. To distinguish between these possibilities, we washed the membrane pellet with an alkaline sodium carbonate solution (0.1 M Na_2_CO_3_, pH 11.0) which removes peripheral membrane proteins (17). Following this wash, both CerR and bCerS were found in the soluble fractions (Fig. 4*B*), consistent with peripheral membrane association. Since CerR localizes to the periplasm, we next determined whether it was associating with the inner or outer membrane. Total membrane fractions were separated by centrifugation through a sucrose gradient. The inner and outer membrane fractions were analyzed by SDS-PAGE and CerR was found in the inner membrane fraction (Fig. 4*C*).

### Lipid profiles of the inner and outer membrane

**Figure 5:**
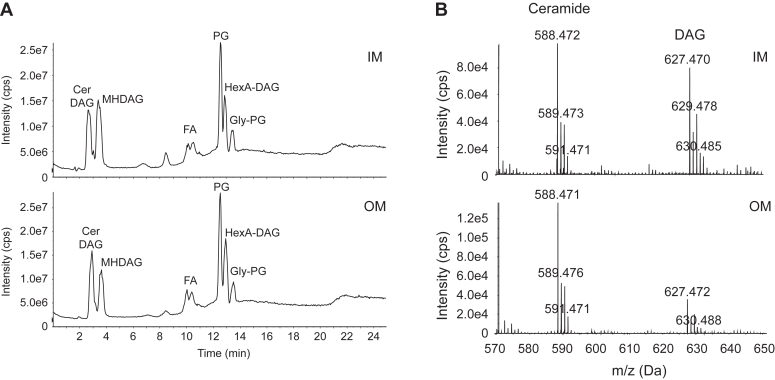
**Lipidomic analysis of inner and outer membrane fractions.** Membrane preparations from *C. crescentus* were separated *via* sucrose gradient centrifugation. The upper (inner membrane) and lower (outer membrane) bands were isolated and then subjected to lipid extraction and LC/MS analysis. *A*, total ion chromatograms of lipids from inner (IM) and outer (OM) membrane fractions show the major lipids identified. *B*, negative ion ESI/MS shows the [M + Cl]^−^ ions of ceramide and DAG species emerging at 2.4 to 4.0 min. Cer, ceramide; DAG, diacylglycerol; FA, fatty acid; Gly-PG, glycyl-phosphatidylglycerol; HexA-DAG, hexuronic acid diacylglycerol; MHDAG, monohexosyl diacylglycerol; PG, phosphatidylglycerol.

Since sphingolipid synthesis begins in the cytoplasm, but the lipids ultimately reach the outer membrane, we used sucrose gradient centrifugation to isolate inner and outer membrane fractions for lipidomic analysis. Total ion chromatographs show no large-scale changes in inner and outer membrane lipid composition (Fig. 5*A*). Because ceramide and diacylglycerol (DAG) co-elute under the normal phase liquid chromatography conditions, we examined the mass spectra of these lipids and found a significantly higher ceramide to DAG ratio in the outer membrane (Fig. 5*B*). These data are consistent with the accumulation of DAG in the inner membrane of *Escherichia coli* (18).

**Figure 6:**
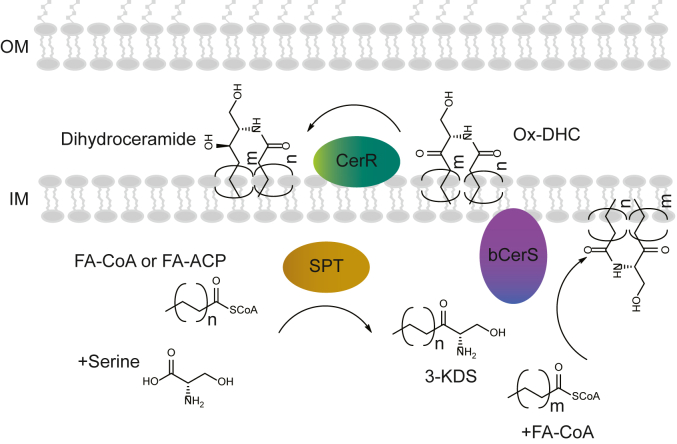
**Model for bacterial sphingolipid synthesis.** Based on our subcellular localization data, we propose that Spt is a soluble cytoplasmic enzyme that condenses either a fatty acid-CoA or a fatty acid-acyl carrier protein with serine to form 3-KDS. bCerS is a cytoplasmic peripheral inner-membrane protein that acylates 3-KDS to oxidized-ceramide/dihydroceramide (DHC). Lastly, CerR is a periplasmic peripheral inner membrane protein that reduces the oxidized sphingolipid to ceramide/DHC. While the synthetic enzymes are well characterized, we have not yet identified any flippases or lipid transporters required to move the sphingolipids across the various membrane compartments.

Based on the protein localization data, together with the buildup of oxidized-ceramide upon *cerR* deletion (6), we propose a model in which Spt is a soluble cytoplasmic protein, bCerS is a cytoplasmic peripheral membrane protein, and CerR is a periplasmic inner membrane-associated peripheral membrane protein (Fig. 6).

## Discussion

Although bacterial sphingolipids were previously thought to be rare, the recent elucidation of the microbial sphingolipid synthesis pathway suggests that these lipids may be present in a wide range of Gram-negative bacteria and smaller groups of Gram-positive organisms (6). The physiological functions of these lipids appear to be species-specific. For example, *Bacteroides thetaiotaomicron* and *Porphyromonas gingivalis* use sphingolipids to suppress host inflammatory processes (2, 19), *Sphingomonas* species replace lipopolysaccharide with glycosphingolipids (7), and *C. crescentus* sphingolipids protect against phage infection (4).

Further study of the physiological roles of bacterial sphingolipids will require a deeper understanding of the biochemical processes involved in their synthesis. Despite the discovery of the genes required for microbial sphingolipid production (5, 6), there is disagreement regarding the order and nature of the biochemical reactions. One model suggests that the synthesis follows the same pathway as that of eukaryotes (5), where the reduction of 3-KDS precedes the addition of a second acyl-chain. Our model proposed that the second acyl chain is added prior to the reduction of the ceramide molecule (6). The data presented in this study aim to distinguish between these models. Based on independent assays of subcellular localization, we have demonstrated that Spt and bCerS are cytoplasmic while CerR is periplasmic (Figs. 2 and 3). This spatial organization is consistent with our model that acylation occurs before lipid reduction. The alternative model would require that 3-KDS (the product of Spt) be translocated to the periplasm for reduction, the resulting sphinganine then be flipped back to the cytoplasm for acylation, and the final lipid being flipped one more time for final translocation to the outer membrane. This back-and-forth process for sphingolipid synthesis seems unlikely and is inconsistent with our previous finding that deletion of *cerR* results in the accumulation of an oxidized-ceramide metabolic intermediate (6).

Although multiple lines of evidence place CerR in the periplasm, this introduces a new question regarding the nature of the reducing equivalent. CerR has homology to the NDUFA9 protein, which is a component of mitochondrial Complex I and functions as an NADH-dependent ubiquinone reductase. The NADH binding site is conserved in CerR (6); however, there is no free NADH in the periplasm to catalyze this reaction. Given its peripheral membrane association (Fig. 4*B*) and its homology to NDUFA9, we hypothesize that CerR may interact with either Complex I or another, yet unidentified, protein which enables electron transfer from NADH to oxidized-ceramide. A similar mechanism is used by the periplasmic nitrate reduction enzyme NarG, which receives electrons from NarI, an inner-membrane cytochrome *b* quinol oxidase (20). Further work will be required to identify CerR-interacting proteins.

While these core synthetic enzymes are found in all bacterial species that make sphingolipids, a potential 3-KDS reductase (KDSR) has been identified in *B. thetaiotaomicron* (21). This enzyme (BT_0972) can reduce 3-KDS to sphinganine *in vitro* and overexpression of BT_0972 converts 3-KDS to sphinganine when expressed in *E. coli*. These results suggest that, in this organism, there may be an additional sphingolipid synthesis pathway that runs parallel to that in eukaryotes. Whether this gene is involved in sphingolipid synthesis *in vivo* is not clear since it appears to be essential, and a loss of function mutant was unavailable for these experiments. As we learn more about the physiology of bacterial sphingolipids, we expect there to be many species-specific lipid metabolic pathways that contribute to the rich diversity of microbial sphingolipid molecules.

## Experimental procedures

### Bacterial strains, plasmids, and growth conditions

The specific strains, plasmids, and primers utilized in this study can be found in Tables S1–S3. More information on the strain construction is also available in the Supplementary Materials. For routine culturing of *C. crescentus* wild-type strain NA1000 and its derivatives, bacteria were cultured at 30 °C in peptone-yeast-extract (PYE) medium. *E. coli* strains were grown at 37 °C in Luria-Bertani (LB) medium. Selection antibiotics were added as required at the following concentration: 50 μg ml^−1^ of spectinomycin in broth and agar for *E. coli* and 25 μg ml^−1^ in broth and 100 μg ml^−1^ in agar for *C. crescentus*. Gene expression was induced in *C. crescentus* by the addition of 0.5 mM vanillate or 0.3% xylose.

### Outer membrane permeabilization and imaging

Chloroform-saturated Tris buffer was prepared by mixing 50 mM Tris, pH 7.4 with chloroform (70:30) and shaking the mixture at room temperature for 30 min. mCherry-fusion strains were induced with xylose or vanillate overnight, collected *via* centrifugation (2 min at 6000*g*, 4 °C), and resuspended in an equal volume of the aqueous phase of the chloroform-saturated Tris buffer. Resuspended cells were rocked for 45 min at room temperature and then washed twice in 50 mM Tris, pH 7.4 (*via* centrifugation for 10 min at 5000*g*) to remove residual chloroform. Control cells were treated as above, but incubated in 50 mM Tris, pH 7.4 without chloroform. The permeabilized cells were spotted onto 1% agarose pads for imaging. Fluorescence microscopy was performed on a Nikon Ti-E inverted microscope equipped with a Prior Lumen 220PRO illumination system, CFI Plan Apochromat 100× oil immersion objective (NA 1.45, WD 0.13 mm), Zyla sCMOS 5.5-megapixel camera (Andor), and NIS Elements v, 4.20.01 for image acquisition.

### Beta-lactam resistance assay

The indicated strains were grown ± vanillate overnight to induce expression of the beta-lactamase fusion proteins. Samples of the overnight cultures were streaked onto PYE agar plates containing ±1 mM vanillate and ±50 μg ml^−1^ carbenicillin. Bacterial growth was assessed after 48 h of growth at 30 °C.

### Subcellular fractionation

Strains encoding FLAG-tagged alleles of the sphingolipid synthesis genes were grown in 500 ml PYE overnight with vanillate (0.5 mM). A 1 ml sample of the culture was removed as a total protein sample. The remainder of the culture was centrifuged at 5000*g* for 10 min, the supernatant was discarded, and the pellet was resuspended in 3 ml TE buffer (10 mM Tris, pH 8, 1 mM EDTA) and lysed by 2 to 3 passages through a French press (20,000 psi). The lysed cells were centrifuged at 4 °C at 10,000*g* for 10 min to remove unbroken cells. The supernatant further centrifuged at 4 °C at 200,000*g* for 1 h to pellet the membranes. Following protein concentration determination by BCA assay, the supernatant (soluble proteins), membranes, and total lysates were solubilized in 1× Laemmli buffer and denatured at 90 °C for 5 min.

### Removal of peripheral membrane proteins

Membrane pellets collected by ultracentrifugation were resuspended at a final concentration of 0.1 μg μl^−1^ with either water (control) or an alkaline solution (0.1 M Na_2_CO_3_, pH 11) and incubated for 30 min at 4 °C (17). The sample was centrifuged at 200,000*g* for 1 h at 4 °C. After ultracentrifugation, the protein concentrations of the supernatant (peripheral membrane proteins) and pellet (intrinsic membrane proteins) were determined (BCA assay) and the samples were solubilized in 1× Laemmli buffer and denatured at 90 °C for 5 min.

### Western blotting

Proteins were resolved by SDS-PAGE on a 12% acrylamide gel with 20 μg of protein per well. After transferring proteins to a nitrocellulose membrane, target proteins were detecting using primary antibodies against the FLAG tag (proteintech; 20543-1-AP; 1:1000), BamA (kind gift from Trevor Lithgow, Monash University; 1:50,000; (22)), and PbpX (kind gift from Martin Thanbichler, University of Marburg; 1:1000; (23)). Bands were detected using horseradish peroxidase-conjugated secondary antibodies and ECL reagents (Cytiva) and imaged on a Bio-Rad Chemidoc MP.

### Membrane separation *via* sucrose gradient centrifugation

Separation of inner and outer membranes was done largely as previously described (24). Briefly, 1 l of *C. crescentus* grown in PYE was collected by centrifugation. The pellet was resuspended in 12.5 ml of 0.5 M sucrose, 10 mM Tris pH 7.5. Lysozyme was added to a final concentration of 100 μg ml^−1^ and stirred on ice for 2 min. 12.5 ml of 1.5 mM EDTA was added dropwise, and the solution was stirred for an additional 7 min on ice. The resulting spheroplasts were collected by centrifugation at 11,000*g* for 10 min. The pellet was resuspended in 25 ml of 0.2 M sucrose, 10 mM Tris pH 7.5, 2 mM MgCl_2_ containing 1 μl benzonase and 1× protease inhibitors. The spheroplasts were lysed *via* three passages through a French press homogenizer (10,000 psi). Unbroken cells were removed by centrifugation and membranes were collected from the supernatant by centrifugation at 184,500*g* for 1 h. Membranes were resuspended in 1 ml 20% (w/v) sucrose, 1 mM EDTA, 1 mM Tris pH 7.5 using a Dounce homogenizer. The sucrose gradient was prepared by layering the following solutions in a 13 ml ultracentrifuge tube: 2 ml 73% (w/v) sucrose, 1 mM EDTA, 1 mM Tris pH 7.5; 4 ml 52% (w/v) sucrose, 1 mM EDTA, 1 mM Tris pH 7.5; 1 ml of the resuspended membranes; the remainder of the tube was filled with 20% (w/v) sucrose, 1 mM EDTA, 1 mM Tris pH 7.5. The samples were centrifuged in a swinging-bucket rotor for 23 h at 288,000*g*. The upper (inner membrane) and lower (outer membrane) bands were collected by pipetting and washed in 10 mM Tris pH 7.5 by centrifugation at 184,500*g* for 1 h. The membrane pellets were resuspended in 500 μl of 10 mM Tris pH 7.5 in a Dounce homogenizer and total protein concentration was measured using a BCA assay kit (Thermo Scientific). Aliquots of the membrane samples were removed for Western blots and the remainder was extracted using the Bligh-Dyer method for LC/MS analysis (25).

#### Liquid chromatography/tandem mass spectrometry (LC/ESI–MS/MS)

Lipid analysis by LC/ESI–MS/MS was performed essentially as previously described (26, 27). Briefly, normal phase LC was performed on an Agilent 1200 Quaternary LC system equipped with an Ascentis Silica HPLC column, 5 μm, 25 cm × 2.1 mm (Sigma-Aldrich). The LC eluent (with a total flow rate of 300 μl min^−1^) was introduced into the ESI source of a high resolution TripleTOF5600 mass spectrometer (AB Sciex). The instrumental settings for negative ion ESI and MS/MS analysis of lipid species were as follows: IS = −4500 V; CUR = 20 psi; GSI = 20 psi; DP = −55 V; and FP = −150 V. The MS/MS analysis used nitrogen as the collision gas. Data analysis was performed using Analyst TF1.5 software (AB Sciex).

## Data availability

All of the data for this work is contained within the manuscript.

## Supporting information

This article contains supporting information (4, 6, 28, 29, 30).

## Conflict of interest

The authors declare that they have no conflicts of interest with the contents of this article.
