# Supplementary material for: Spatial organization of bacterial sphingolipid synthesis enzymes
Source: J Biol Chem. 2024 Apr 7;300(5):107276. doi: 10.1016/j.jbc.2024.107276 (PMC11087976; doi:10.1016/j.jbc.2024.107276)
Supplement: Supporting information [file mmc1.pdf]

Supplementary Methods

<u>Strain construction</u>

Strains for vanillate-inducible expression of FLAG-tagged sphingolipid synthesis genes were created by PCR amplifying *spt* (EK1306/1307), *bcerS* (EK1199/1200), and *cerR* (EK1158/1174) from NA1000 genomic DNA and ligating into the NdeI/NheI site of pVCFPC-1. The resulting plasmids were transformed into their respective gene knockout strains.

Strains for vanillate-inducible expression of b-lactamase fusions were created by PCR amplifying the following genes from NA1000 genomic DNA without the terminal stop codon: *spt* (EK1306/1312), *bcerS* (EK1199/1313), and *cerR* (1158/1309). The resulting DNA fragments were ligated into the NdeI/EcoRI site of pVCFPC-1. The beta-lactamase gene, without the first 69 bases encoding the signal sequence, was PCR amplified from pBAD18 (EK1311/1318) and ligated into the EcoRI/NheI site of the respective fusion plasmids. The resulting plasmids were transformed into the Δ*bla6* background.

Supplementary Table 1. Strains used in this study.

Strain	Genotype	Construction	Source
C. crescentus			
NA1000	Synchronizeable variant of wild-type *C. crescentus* strain CB15		(1)
GS32	Δ*ccna_01220* (*spt*)		(2)
GS140	Δ*ccna_01222* (*cerR*)		(3)
GS198	Δ*ccna_01212* (*bcerS*)		(3)
CU19	Δ*ccna_01220*; P*vanA*:: *ccna_01220-FLAG*	Transformation of GS32 with pCU16	This study
CU24	Δ*ccna_01222*; P*vanA*:: *ccna_01222-FLAG*	Transformation of GS140 with pCU22	This study
GS215	Δ*ccna_01212*; P*vanA*:: *ccna_01212-FLAG*		(3)
EK291	P*xylX*:: *gspG-mCherry*		(3)
EK308	P*xylX*:: *TAT-mCherry*		(3)
GS289	P*vanA*:: *ccna_01220-mcherry*		(3)
GS290	P*vanA*:: *ccna_01222-mcherry*		(3)
GS291	P*vanA*:: *ccna_01212-mcherry*		(3)
LS177	Δ*bla6*		MRK Alley (unpublished)
CU65	*Δbla6* P*vanA*:: *ccna_01220-bla*	Transformation of LS177 with pGS281	This study
CU70	*Δbla6* P*vanA*:: *ccna_01222-bla*	Transformation of LS177 with pGS282	This study
CU71	*Δbla6* P*vanA*:: *ccna_01212-bla*	Transformation of LS177 with pGS283	This study
CU75	*Δbla6* P*vanA*:: *ccna_01220-FLAG*	Transformation of LS177 with pCU16	This study
CU77	*Δbla6* P*vanA*:: *ccna_01222-FLAG*	Transformation of LS177 with pCU22	This study
CU76	*Δbla6* P*vanA*:: *ccna_01212-FLAG*	Transformation of LS177 with pGS193	This study
E. coli			
DH5a	Cloning strain		Thermo Scientific

Supplementary Table 2. Plasmids used in this study.

Name	Description	Source
pVCFPC-1	Vanillate-inducible expression, $Spect^R$	(4)
pGS193	pVCFPC-1-based plasmid for *ccna_01212-FLAG* expression, $Spect^R$	(3)
pCU16	pVCFPC-1-based plasmid for *ccna_01220-FLAG* expression, $Spect^R$	This study
pCU22	pVCFPC-1-based plasmid for *ccna_01222-FLAG* expression, $Spect^R$	This study
pBAD18	Source of beta-lactamase gene (*bla*)	(5)
pGS281	pVCFPC-1-based plasmid for *ccna_01220-bla* expression, $Spect^R$	This study
pGS282	pVCFPC-1-based plasmid for *ccna_01222-bla* expression, $Spect^R$	This study
pGS283	pVCFPC-1-based plasmid for *ccna_01212-bla* expression, $Spect^R$	This study

Supplementary Table 3. Primers used in this study.

Name	Sequence
EK1158	tactcatATGGCGACTGACGCGCGC
EK1174	tactgctagcTTActtgtcatcgtcatccttgtagtcGGCGACGATATTTTTGGGTAGCC
EK1199	tactcatATGCCTTTCGACTCCACCAACG
EK1200	tactgctagcTTActtgtcatcgtcatccttgtagtcCAGGGCCTTCTCGTAGACGC
EK1306	tactcatATGGGGCTATTTGATAAGCACCTGG
EK1307	tactgctagcTTActtgtcatcgtcatccttgtagtcGGCGCGGGCGCGCTTGAG
EK1309	tactgaattcGGCGACGATATTTTTGGGTAGCC
EK1311	tactgctagcctaCCAATGCTTAATCAGTGAG
EK1312	tactgaattcGGCGCGGGCGCGCTTGAG
EK1313	tactgaattcCAGGGCCTTCTCGTAGACGC
EK1318	tactgaattcggtggaggtggatctCACCCAGAAACGCTGGTGAAAG

Supplementary references

1. Nierman, W. C., Feldblyum, T. V., Laub, M. T., Paulsen, I. T., Nelson, K. E., Eisen, J. A., Heidelberg, J. F., Alley, M. R., Ohta, N., Maddock, J. R., Potocka, I., Nelson, W. C., Newton, A., Stephens, C., Phadke, N. D., Ely, B., DeBoy, R. T., Dodson, R. J., Durkin, A. S., Gwinn, M. L., Haft, D. H., Kolonay, J. F., Smit, J., Craven, M. B., Khouri, H., Shetty, J., Berry, K., Utterback, T., Tran, K., Wolf, A., Vamathevan, J., Ermolaeva, M., White, O., Salzberg, S. L., Venter, J. C., Shapiro, L., and Fraser, C. M. (2001) Complete genome sequence of *Caulobacter crescentus*. *Proc Natl Acad Sci U S A* **98**, 4136-4141
2. Stankeviciute, G., Guan, Z., Goldfine, H., and Klein, E. A. (2019) *Caulobacter crescentus* adapts to phosphate starvation by synthesizing anionic glycoglycerolipids and a novel glycosphingolipid. *mBio* **10**, e00107-00119
3. Stankeviciute, G., Tang, P., Ashley, B., Chamberlain, J. D., Hansen, M. E. B., Coleman, A., D'Emilia, R., Fu, L., Mohan, E. C., Nguyen, H., Guan, Z., Campopiano, D. J., and Klein, E. A. (2022) Convergent evolution of bacterial ceramide synthesis. *Nat Chem Biol* **18**, 305-312
4. Thanbichler, M., Iniesta, A. A., and Shapiro, L. (2007) A comprehensive set of plasmids for vanillate- and xylose-inducible gene expression in *Caulobacter crescentus*. *Nucleic Acids Res* **35**, e137
5. Guzman, L. M., Belin, D., Carson, M. J., and Beckwith, J. (1995) Tight regulation, modulation, and high-level expression by vectors containing the arabinose PBAD promoter. *J Bacteriol* **177**, 4121-4130
